# Estimating the Orientation of Objects from Tactile Sensing Data Using Machine Learning Methods and Visual Frames of Reference

**DOI:** 10.3390/s19102285

**Published:** 2019-05-17

**Authors:** Vinicius Prado da Fonseca, Thiago Eustaquio Alves de Oliveira, Emil M. Petriu

**Affiliations:** School of Electrical Engineering and Computer Science, University of Ottawa, Ottawa, ON K1N 6N5, Canada; talvesde@uottawa.ca (T.E.A.d.O.); petriu@uottawa.ca (E.M.P.)

**Keywords:** in-hand manipulation, pose estimation, machine learning, fuzzy control, underactuated robotic hands

## Abstract

Underactuated hands are useful tools for robotic in-hand manipulation tasks due to their capability to seamlessly adapt to unknown objects. To enable robots using such hands to achieve and maintain stable grasping conditions even under external disturbances while keeping track of an in-hand object’s state requires learning object-tactile sensing data relationships. The human somatosensory system combines visual and tactile sensing information in their “What and Where” subsystem to achieve high levels of manipulation skills. The present paper proposes an approach for estimating the pose of in-hand objects combining tactile sensing data and visual frames of reference like the human “What and Where” subsystem. The system proposed here uses machine learning methods to estimate the orientation of in-hand objects from the data gathered by tactile sensors mounted on the phalanges of underactuated fingers. While tactile sensing provides local information about objects during in-hand manipulation, a vision system generates egocentric and allocentric frames of reference. A dual fuzzy logic controller was developed to achieve and sustain stable grasping conditions autonomously while forces were applied to in-hand objects to expose the system to different object configurations. Two sets of experiments were used to explore the system capabilities. On the first set, external forces changed the orientation of objects while the fuzzy controller kept objects in-hand for tactile and visual data collection for five machine learning estimators. Among these estimators, the ridge regressor achieved an average mean squared error of $0.077^{\circ }$. On the second set of experiments, one of the underactuated fingers performed open-loop object rotations and data recorded were supplied to the same set of estimators. In this scenario, the Multilayer perceptron (MLP) neural network achieved the lowest mean squared error of $0.067^{\circ }$.

## 1. Introduction

Widespread in controlled environments such as industries, robots are moving towards unstructured settings like homes, schools, and hospitals performing high-level, complex, and fast reasoning; nevertheless, several challenges remain unsolved for robot skills achieving human-level capabilities [1]. Although robots can accurately perform several tasks such as walk, pick and place objects, understand, and communicate with people, they still present a lack of hand dexterity. Improvements on tactile sensing for in-hand manipulation and an increased understanding of human perception to action inspire and could advance robot possibilities in the scenarios mentioned above [2].

Manipulation skills developed in the human’s hand and the brain have a level of ability rarely seen in other animals. Grasping and manipulating objects is a distinctive part of the human-being skill set. It is an ability evolved from the erect posture that freed our upper limbs, turning our hands into two sophisticated sets of tools [3]. Not surprisingly, humans’ hand dexterity and reasoning are the holy grail of bio-inspired robotics control and actuation. Hand dexterity is the ability to interact in a useful way with objects in the real world. During robotic manipulation, a robot changes an object’s state from an initial configuration to a final pose. For instance, during pick and place tasks, the goal of robotic platforms is to change the position and orientation of an object inside the manipulator’s workspace. Comparatively, in-hand manipulation is the ability to change the pose of objects, from initial orientation to a given one, within one hand. Implementing robots that change objects’ orientation while maintaining a stable grasp has the potential to amplify even more robot area of activity [4]. Robotic manipulator literature comprises a long list of examples of robotic hands with many levels of dexterity. From there, underactuated hands arise as an option that can achieve a reasonable level of dexterity with simplicity.

Research on haptic perception over the past decades has developed an in-depth knowledge of the psychological aspects of human touch employed to manipulate objects and perceive their characteristics. Lederman et al. [5] presented the somatosensory system divided into two subsystems: The “What” system that carries out perception and memory; and the “Where” system that deals with perception to action. In humans, the “What” system performs the recognition of surfaces and objects through their tactile properties [6]. Robots that implement similar recognition systems demonstrate their efficacy when similar objects are identified promptly even without visual feedback [7]. From this perspective, Reference [8] is one example in recent research, where authors employed camera-based sensing and deformable material as input to an object recognition system integrating grasping and object recognition. On the other hand, the “Where” system, which has a counterpart in vision, produces a description of points, surfaces, and reference frames in the world. Different from vision, touch refers to a location in the sensory organ, the skin itself, and localization in the environment. The human sensorial loop combines tactile perception in the presence of vision had attracted research interest due to increased reliability when both modalities [9]. Recent literature presents several approaches to this issue with a significant contribution on the tactile feedback topic were inspired by the concept of the tactile flow in humans, and improvements have been studied on the analysis of tactile feedback in robots using computational tactile flow [10]. Although our work is also inspired by the human somatosensory system, we took different approaches for a similar goal. Instead of using the human tactile flow as a base for our research, we developed pose estimation based on the human visuotactile system, also called the “Where” system. This intersensory interaction extends to situations combining vision and touch to include different object information, for interobject interaction or allocation of attention [5].

During visuotactile interaction, multiple frames of reference are simultaneously available on human haptic spatial localization [5]. In the “Where” system, two types of touch spatial localization are considered, one being a position on the body where the stimulus is applied, or otherwise, in the external world from where the stimulus comes. During a task, humans can use a single frame or combine multiple frames of reference, the origins of which may be visible landmarks or a body part from the individual. Even though the aforementioned haptic frame describes the contact between the skin and an external object, one could use landmark axes to specify a frame of reference external to the body (an “allocentric” frame of reference). Similarly, a local frame of reference, such as a fingertip axis, is used for localization on the body (an “egocentric” frame of reference). In summary, haptic spatial localization could be described either by a coordinate system centered on the body, egocentric or by external features in the environment such as the edges of a table, an allocentric frame of reference.

Investigations on tactile sensing have the potential to improve robotic in-hand manipulation, including, but not limited to, object characteristics extraction and feedback control. Tactile sensing provides essential information about object manipulation, solving problems such as object occlusion and object’s pose estimation under stable grasping. Form, shape, and functionality of the human skin also inspired research on the tactile sensing field. A bio-inspired approach to tactile sensing has significant results in the literature, including successful works on texture classification and control feedback. The present work uses a visuotactile approach to pose estimation using data collected from bio-inspired multimodal tactile modules in conjunction with camera feedback.

Successful robotic manipulation starts with a stable object grasp; therefore, robots are expected to have robust grasping skills. Considering grasp as a control problem, in contrast to decomposing a grasping procedure into planning and execution, they do not require any specific hand–object relative pose and are more robust under pose uncertainty [11]. A model-free solution in addition to computationally inexpensive control laws uses simpler hand designs, still ensuring a stable grasping solution. Fuzzy logic control for grasp stability is present in the literature as a useful tool for in-hand manipulation [12,13,14,15]. Multifingered hands achieve stable gasping when no resultant forces act on a fully restrained object [16]. This work was developed using a fuzzy controller that was able to perform stable grasp tasks with controlled fingertip force single and dual actuated versions.

The main contributions of this paper are: (1) Tactile information to estimate the pose of unknown objects under autonomous, stable grasp; (2) Integration of bio-inspired multimodal tactile sensing modules and visual information to describe in-hand object pose; (3) Analysis of five different machine learning algorithms for tactile pose estimation. The system uses visual information similar to how humans allocate visual attention to determine frames of reference for objects of interest. Tactile information is used to learn vision-defined reference frames so that the vision system can be freed to perform other tasks [5]. Concretely, we used vision to extract an allocentric frame of reference where the object pose is located in the environment. Further, five machine learning methods using data from the tactile sensing system inferred the relation between egocentric and allocentric reference frames during haptic spatial localization. Post-grasp object rotations were performed to collect tactile information, exposing the learning system to object angles outside of the finger’s actuation workspace. For this purpose, the object received external forces in two different axes, including an open-loop finger actuation experiment. A closed-loop stable grasp fuzzy controller also used the same sensory feedback signals. From all six machine learning algorithms, results using ridge regressor achieved an average mean squared error for all sizes of 1.82°.

In this paper, Section 2 presents a literature review of human haptic perception, in-hand robotic manipulation, tactile sensing, fuzzy control, and regression algorithms. Section 3 presents our prototype description, a system overview, and experimental setup. Section 4 shows experimental results for two in-hand manipulation tasks performed, followed by conclusions in Section 5.

## 2. Literature Review

Several papers in recent years have approached the robotic grasping and manipulation in its different aspects. Some of them are design, control, and sensing. The present work approaches in-hand manipulation aspects of control and sensing using the human “Where” sensory system as an inspiration to in-hand object pose estimation.

### 2.1. The Human “Where” System

Studies on the human somatosensory divided it into “What” and “Where” systems related to the functional distinction between them, the former being the system that processes surfaces, objects, and their characteristics, while the latter being responsible for describing points, surfaces, and objects in the environment. With a counterpart in vision, the “Where” system is investigated in the present paper as an approach to solve in-hand manipulation pose estimation of objects. A notable difference from vision, however, is that the localization can be related to the sensory organ, the human skin, or to the world. Accordingly, investigations focus on two types of visual–spatial localization on the human tactile field, one determining where on the body a stimulus is being activated, and another wherein the external world a body tactile sensing is being touched [5].

In order to study human touch as a spatial localization, researchers require investigation of how humans specify frames of reference [9]. A frame of reference defines Cartesian or polar coordinate systems, where its origins may be an individual’s body parts or distinct points to build environment landmarks. Humans can use a single frame of reference to perform a given task, but multiple frames of reference are available. An example from [5] defines points of the body, such as fingertip axes, that act as local frames of reference. When facing the task of localizing points in the external space, humans usually refers to an “egocentric” frame of reference, which specifies distances and directions relative to the individual. By contrast a person uses an “allocentric” frame of reference when using landmarks and external axes as guidelines. The ability to use multiples frames of reference is also essential to the allocation of attention that human dexterity is known. Higher-level visual-touch interactions are a great example of how the human somatosensory system uses those frames of reference to collect information about different objects, during interobject interaction or allocation of attention.

### 2.2. Manipulation, Dexterity, and Underactuated Hands

Biological inspiration in robotics has been growing over the years with significant interaction between robotics and neuroscience, even though the neuroscience background is usually an endless inspiration with small impact on the final application [3]. Recent research on visuotactile grasping and manipulation has shown that robots have improved manipulation skills when simulating human tactile and visual perception to action. Bimbo et al. [17] combined vision and touch for the estimation of an object’s pose during in-hand manipulation where the camera suffers from finger occlusion. A visuotactile control was developed by Li et al. [18], achieving robust in-hand manipulation and exploration of unknown objects performing robust manipulation even in the presence of accidental slippage or rolling. [19] presents another example of reliable grasps using underactuated hands, where flexible hands and visual clues were used to perform after grasping pose estimation for high-precision assembling.

Adaptive hands, such as those used in the works mentioned above, have become an exciting topic of research over the last decade, with recent development in several areas, such as new designs [20,21], reliable grasping [12,22], and dexterous in-hand manipulation [23,24]. Due to its unique ability to grasp and adapt to unknown objects, special attention has been devoted to the research of underactuated hands in unstructured environments. This characteristic is because underactuated hands have *n* Degrees of Freedom (DOF), and fewer than *n* actuators, while kinematic constraint of the finger is promoted by passive elements, e.g., springs of flexible joints, establishing the shape adaptation of the finger to the object grasped. Dynamics of underactuated hands also have outstanding results with simpler solutions, using tactile sensing with force control on top of slippage detection [25]. Odhner et al. [21] described a design where power and precise grasping are made possible by flexible joints and two pivoting fingers. Reasonable dexterity was achieved by having opposed fingers with individual actuators and tactile feedback provided by its force sensors. Even though little space for customization is observed in the solution as mentioned above, Zisimatos et al. [20] designed a single actuated robotic gripper that supported up to six modular fingers pulled by a differential disk. Later, we redesigned the base and finger to enable dual actuation for opposed fingers which, in conjunction to modified phalanges, made mounted multimodal tactile sensing modules possible with similar dexterity. Shortly, the present paper uses a prototype that falls in between the examples as mentioned earlier with a modified version of [20]’s fingers and base, using two actuators, borrowing ideas from [21].

### 2.3. Tactile Sensing

Although manipulation tasks could use several types of cameras and noncontact sensors for feedback, tactile sensing plays a vital role in providing accurate local data for intelligent control and manipulation [26]. With tactile data and feedback, the object’s pose can be inferred using statistical and intelligent techniques [27,28]. Due to its importance in manipulation, tactile sensors have had massive development in recent years. In the literature, tactile sensors could use different technologies, such as piezoresistive, capacitive, piezoelectric, optical barometric-based, sound, and multimodal sensing [29]. Among them, multimodal techniques provide a capability that closely matches the human skin, itself a multimodal sensing organ.

**Figure 1:**
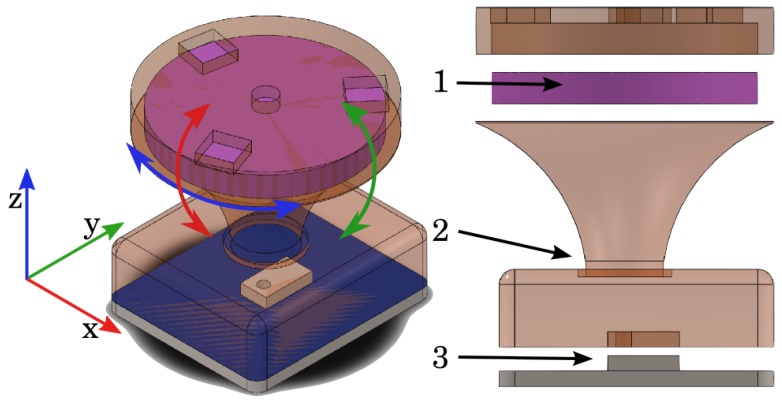
Components of the tactile sensing module: 1—MARG (magnetic, angular rate, and gravity) system; 2—compliant structure; 3—barometer [30].

Alves de Oliveira et al. [30] developed a bio-inspired multimodal sensor module observing the functionality and placement of mechanoreceptors in the human skin. Figure 1 presents the sensing module organization where a compliant cone-like structure (2) supports a magnetic, angular rate, and gravity (MARG) system (1) and guides forces acting on the module’s surface to the pressure sensor localized on the bottom of the structure. In addition to force, the inertial measurement provides information about the deformation of the artificial skin when pressure is applied.

This paper presents an application of bio-inspired tactile sensing modules and a visuotactile approach used in addition to machine learning algorithms for in-hand object pose estimation. Similar to the human somatosensory system, information about the skin itself provides an ‘egocentric’ frame of reference where the object’s interaction to the artificial skin occurs. Real-time information obtained by the tactile modules was also used to maintain a stable grasp during experiments with an underactuated robotic hand.

### 2.4. Fuzzy Stable Grasping

Using fuzzy logic to address the grasping problem is one way to reduce the gap between conventional and intelligent control due to the uncertainties and complex mathematical solutions involved in the task [31]. In a recent edition of the Amazon picking challenge, the authors of [32] described problems with an open-loop control solution during pick and place tasks; therefore, autonomous, stable grasping is an essential aspect of in-hand manipulation. The authors of [33] presented a closed loop control using tactile sensing for a task of removing a book from a bookshelf when maximization of the contact surface is required.

Another aspects investigated by [12] are relations between microvibrations and grasp stability. The authors developed a dual motor fuzzy control using BioTac^©^ sensor feedback. Grasping status and stability values were estimated using pressure and microvibration data. A second fuzzy controller provides finger directives such as pull, push, or hold using the previous stability values. In multifingered hands, when no resultant force acts on a fully restrained object, equilibrium is achieved, which is a requirement for stable grasp [16]. The present work implemented a fuzzy controller that was able to perform grasping tasks with minimum fingertip force. Our controller used microvibrations, detected from the sensor’s gyroscope and force from fingertips sensor to control each finger separately. A previous version also studied used an Force-sensing resistor (FSR) sensor providing input to a single motor fuzzy controller.

The next section presents an experimental setup for our visuotactile experiment in addition to details about the prototype used, material, and methods approach.

## 3. Materials and Methods

**Figure 2:**
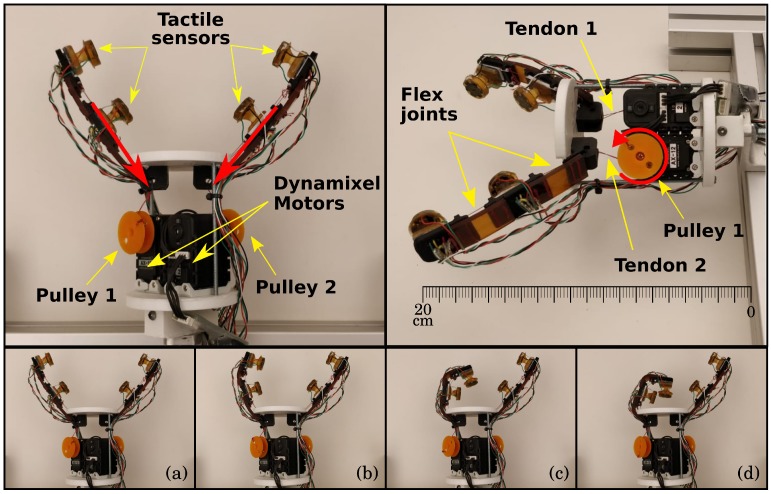
Top and side view of the modified gripper design. (**a**) rest position; (**b**) initial movement; (**c**) finger actuation; (**d**) maximum safe curvature.

This section presents materials, methods, and experimental setup for visuotactile object pose estimation. The open source design from [20] is the basis for the present implementation. It is a modular 3D printed gripper in ABS plastic providing an easily customizable platform. Our robotic hand design is a two independently controlled fingers version mounted on top of a table. For that goal, we kept Zisimatos et al.’s [20] top plate, but a modified base accommodates two motors needed to pull each finger separately. When compared to the human hand, these underactuated robotic fingers have intermediate and distal flexible joints made of Vitaflex^©^ 30 (Smooth-On, Easton, PA, USA). Based on Zisimatos et al. [20], this top plate, including the fingers, shows a maximum force applied (and retained) of 8 *N* per fingertip during tests with a standard servo. Strings are attached to tip phalanges, and two fingers are pulled independently by two Dynamixel^©^ motors (Robotis, Lake Forest, CA, USA) mounted on a modified base. From base to fingertip, the gripper is about 20 cm long. Figure 2 shows the opened gripper during experiments before a grasp attempt. At the top, the left picture shows the four tactile sensors mounted on the finger phalanges, motors, and pulleys. This viewpoint is used to place the camera, as shown in Section 3.3. The red arrows indicate the direction of force applied by the motors. On the top right of Figure 2, a side view shows details of tendons and flex joints. A detail of the pulley one appears with a red circular arrow indicating the motor actuation direction during the pulling phase. The bottom row of Figure 2 shows steps of a single finger actuation: (a) Rest position, no motor rotation; (b) initial movement with motor pulling; (c) continuous motion brings the finger to closer to the palm; and (d) around the maximum safe finger curvature.

Each finger phalange has a tactile sensor module mounted on it. Figure 2’s top row shows the tactile module multimodal sensors and its placement on each finger. The module’s compliant structure and material add flexibility for the fingers’ functionality. All tactile modules send data to microcontrollers acting as nodes to the central computer. The software developed for this prototype is a distributed system that uses Robot Operating System (ROS) [34].

**Figure 3:**
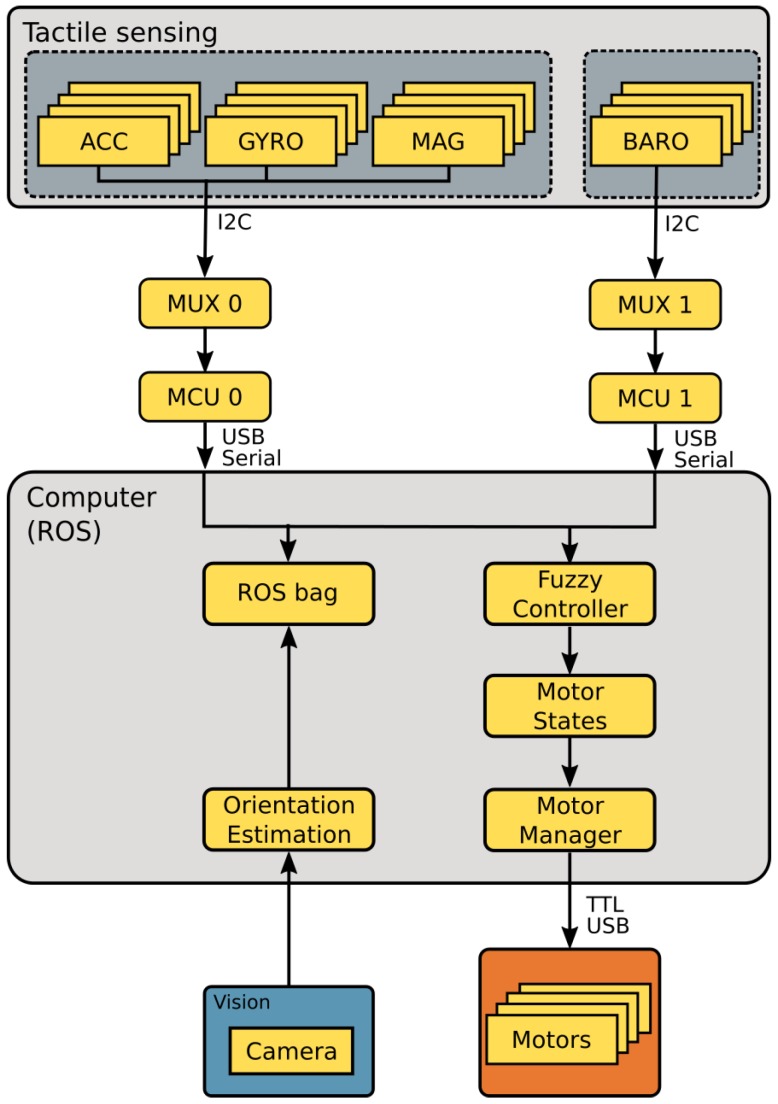
System overview.

Figure 3 presents all primary ROS nodes developed for this implementation in yellow boxes inside the “Computer (ROS)” gray box. The central node runs in a laptop that concentrates control, data collection, and pose estimations. All data were collected to *ROS bags* for post-processing and pose orientation estimation. Figure 3 also shows the fuzzy control node that also receives sensor data and updates motor controllers. MCU 0 and MCU 1 are microcontrollers attached via serial connection to a main computer acting as ROS nodes. These microcontrollers receive data from magnetic, angular rate, and gravity (MARG) and barometer components of via I^2^C protocol that is represented by arrows from the “Tactile sensing” module in Figure 3, which also shows I^2^C communication from tactile modules multiplexed to via MUX 0 and MUX 1. There is also a USB camera represented in a blue box and a USB Serial connection from the “Motor Manager” yellow box used for computer control of Dynamixel motors represented in an orange box at the bottom of Figure 3. In the following sections, we present tactile sensing module components and organization as well as the fuzzy control system used in this experimental setup.

### 3.1. Tactile Sensors

Bio-inspired multimodal tactile sensing modules which are inspired by the type, functionality, and organization of cutaneous tactile elements provide tactile information during visuotactile perception experiments. Each module contains a 9 DOF, magnetic, angular rate, and gravity (MARG) sensor, a flexible, compliant structure, and a pressure sensor placed in a structured way similar to human skin [30]. The components integrated in the tactile module are previously discussed in Section 2 and shown in Figure 1. A total of four tactile modules, one for each phalange, had data collected during experiments. Figure 3 shows four MARGs connected to microcontroller MCU 0 by a multiplexer MUX 0. In a similar manner, four deep pressure sensors are connected to microcontroller MCU 1 by a multiplexer MUX 1. The master ROS node running at the central computer demultiplexes the data and stores tactile information represented in Figure 3 by arrows from the USB serial connection to a yellow box labelled “ROS bag”. The experiments and results section presents data provided by the tactile modules during the execution of rotation using both external forces and open-loop execution of in-hand manipulation.

### 3.2. Fuzzy Controllers

Before any manipulation took place, two fuzzy controllers based on tactile sensing information such as microvibrations and pressure maintained a stable grasp by sending motor signals to each actuator responsible for pulling the fingers [12]. The autonomous fuzzy grasping controller system on this setup provided consistent grasp force while handling different object sizes.

**Figure 4:**
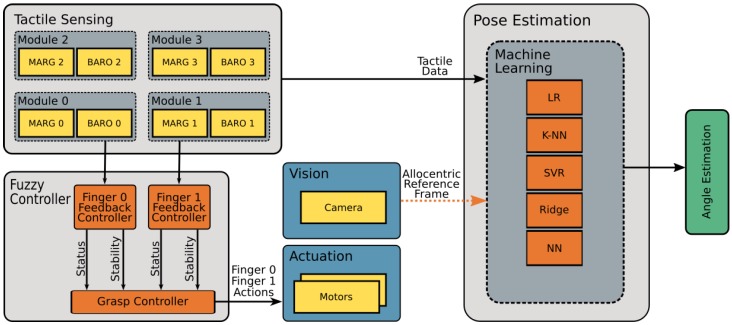
System flow chart.

The present implementation used a dual fuzzy controller based on pressure and microvibrations. The pressure is measured from the deep pressure sensor readings, while gyroscope information provides microvibration values for a fuzzy feedback controller about each finger. The indicator that the object under grasp has movement is angular velocity used here to detect microvibrations. The pressure sensor shows the degree of contact with the object. In conjunction, angular velocity and pressure provided tactile feedback about stability and status to a second fuzzy grasp controller. Two gray boxes named *Tactile module 0* and *Tactile module 1* in Figure 4 describe barometer and MARG components with separated I^2^C signals forwarded to independent finger fuzzy feedback controllers.

Figure 4 describes a complete system flow during the experiments. Data from tactile sensing modules (top left) provided information for the fuzzy controller (bottom left) with details described in the following sections. With a grasp controller decision based on stability and status, finger actions modified the actuators’ status (blue box middle). Vision (middle blue box) provides an allocentric reference frame to the machine learning pose estimation. This allocentric reference frame used data from all four tactile modules to provide an egocentric frame of reference, which was input to the pose estimation module (right gray box). The last step was to apply five machine learning techniques and return angle estimation (green box).

Each *finger fuzzy feedback controller* provided status and stability values to a *grasp fuzzy controller*, which is indicated by a gray box inside the *fuzzy controller* box receiving status and stability inputs in Figure 4. The *grasp fuzzy grasp controller* produced actions for each finger (go forward, go backward or hold) based on status and stability information from both fingers.

#### 3.2.1. Finger Fuzzy Feedback Controller

The *finger fuzzy feedback controller* used real sensor data to provide pressure status and grasp stability information for input to a *grasp fuzzy controller*. In order to describe finger fuzzy feedback controller inputs, Low and High fuzzy sets were defined for microvibrations input and Nopressure, Lowpressure, Normalpressure, and Highpressure fuzzy sets for pressure input. Stable and Notstable fuzzy sets describe finger fuzzy feedback stability output while Nottouching, touching, Holding, and Pushing define status output.

**Figure 5:**
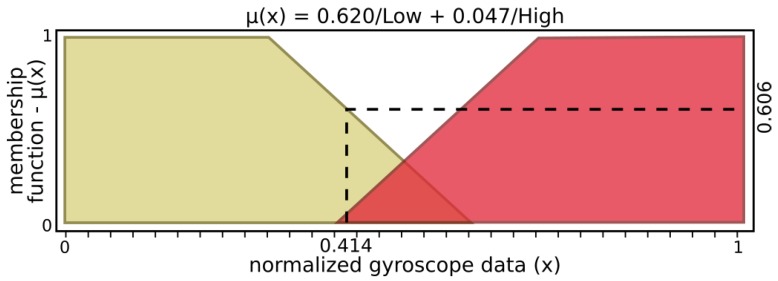
Input sets of microvibrations.

**Figure 6:**
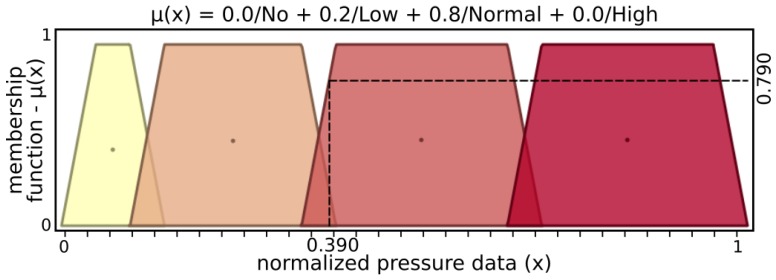
Input sets of pressure.

**Figure 7:**
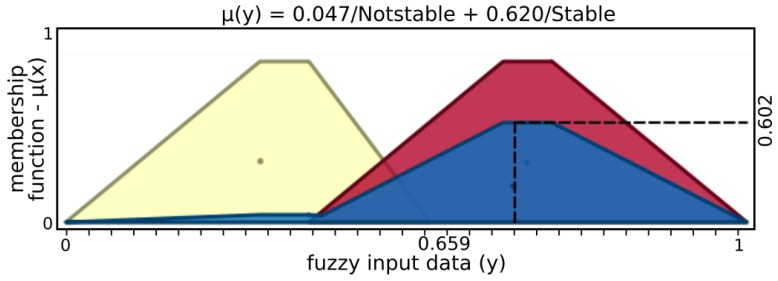
Output sets of stability.

**Figure 8:**
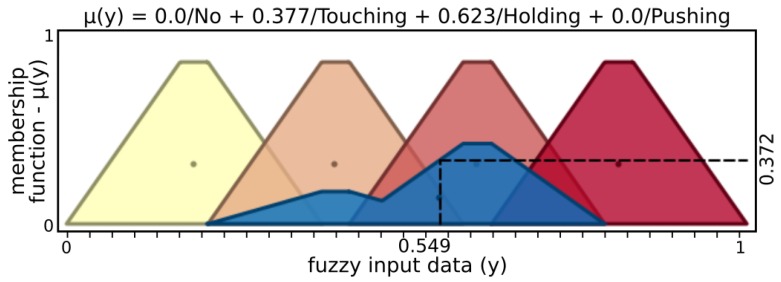
Output sets of status.

Figure 5 and Figure 6 present input values applied for microvibrations and pressure fuzzy sets, respectively. The tactile information used here was normalized data from the barometer part of the tactile module as pressure and raw gyroscope data measuring variations on the angular velocity of the respective module. An example of possible output, Figure 7 and Figure 8 show stability and status output form its fuzzy sets, respectively. The inference system used was Mandani based on the center of gravity. The second fuzzy controller used status and stability from this both finger’s controller to produce finger actions.

A rule book based on [12] was implemented for this tactile in-hand manipulation fuzzy feedback controller, and Table 1 summarizes the rules. Possible outputs for status are NT, not touching; T, touching; H, holding; and P, pushing. Stability has two possible outputs, S, stable and NS, not stable.

From the example above, microvibration data activate 0.620 of “Low” and 0.047 of “High” sets, resulting in μ(x)=0.606, where μ(x) is a membership function. In a similar way, the above example of pressure has μ(x)=0.790 with 0.2 of “Low” and 0.8 “Normal” pressure sets, while “No” and “High” sets have no participation. After inference, stability presents μ(y)=0.602, activating 0.620 of “Stable” and 0.047 of “Nonstable”, while status has μ(y)=0.372, with 0.377 of “Touching” and 0.623 of “Holding”.

#### 3.2.2. Grasp Fuzzy Controller

**Figure 9:**
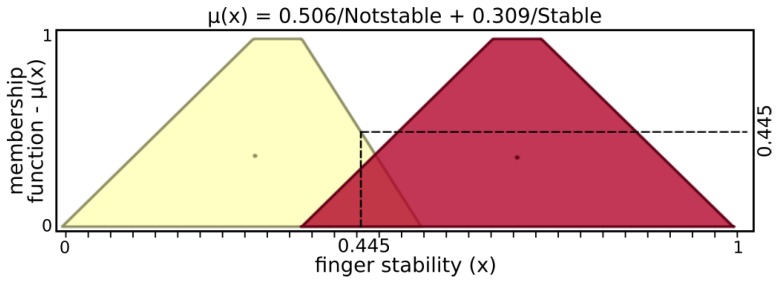
Input sets of stability.

**Figure 10:**
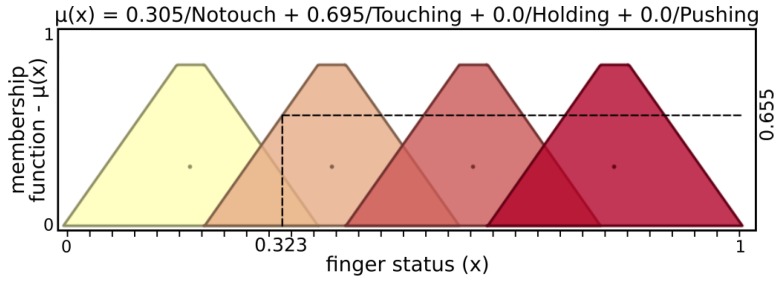
Input sets of status.

**Figure 11:**
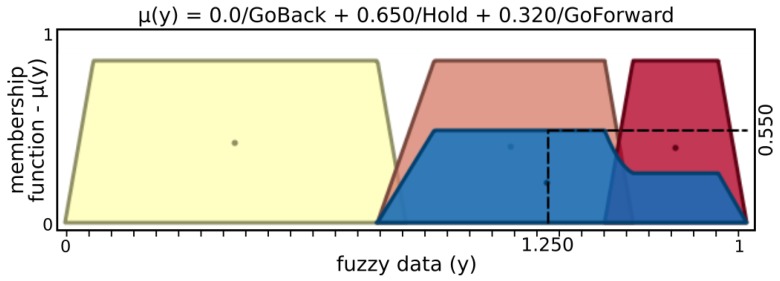
Action output sets.

Based on both finger status and stability, a second fuzzy controller is responsible for publishing motor values in order to maintain a stable grasp. The system overview in Figure 4 shows a box labeled grasp fuzzy controller, where stability and status data from each finger are inputs for this fuzzy controller, while its outputs update motor velocities. Above, *finger fuzzy feedback controller* provided status and stability from each finger. It is important to observe that input from both fingers is essential during the inference phase of the *grasp fuzzy controller*. Although both fingers are necessary for inference, for simplicity, Figure 9 and Figure 10 only present one finger input example. The inference system used was also Mandani based on the center of gravity. Both outputs change finger motor velocity, updating the Dynamixel controller manager node such as presented on Figure 11, where Figure 9 and Figure 10 show sets that define inputs of finger stability and status, respectively.

Another rule book also based on [12] was implemented for the grasp fuzzy controller and is summarized in Table 2. Possible outputs for updating motor velocities are GF1, finger 1 go forward; GF2, finger 2 go forward; H1, finger 1 hold; H2, finger 2 hold; and GB1, finger 1 go back; GB2, finger 2 go back.

From the example above, stability inputs provide μ(x)=0.445, with 0.506 of “NotStable” and 0.309 of “Stable”. Similarly, status input results in μ(x)=0.655, with sets Notouch of 0.305 and Touching of 0.695, while Holding and Pushing are not activated. After inference, the grasp fuzzy controller produces μ(y)=0.550, with contributions of 0.650 from “Hold” and 0.320 from “GoForward”.

**Figure 12:**
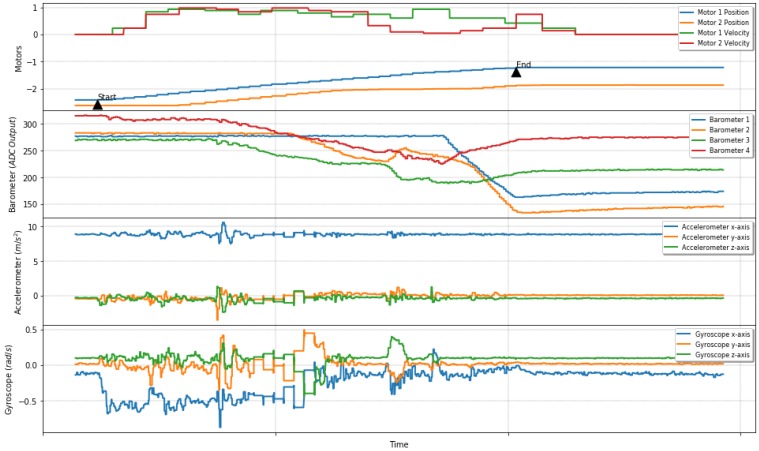
Actuator velocities during a stable grasping. Barometers, accelerometers, and gyroscopes values varying until stable grasp, followed by stabilization of sensor values.

Figure 12 shows sensor data, motor positions, and velocities during an autonomous grasp attempt. Start and end arrows point the beginning and final phases of a grasping attempt. Motor velocities and tactile sensing data are fast changing during those two points. Straight lines after the end arrow denote that sensor data and motor position achieve stability, and the fuzzy controller is maintaining a stable grasp between a reasonable error margin.

### 3.3. Camera Setup

**Figure 13:**
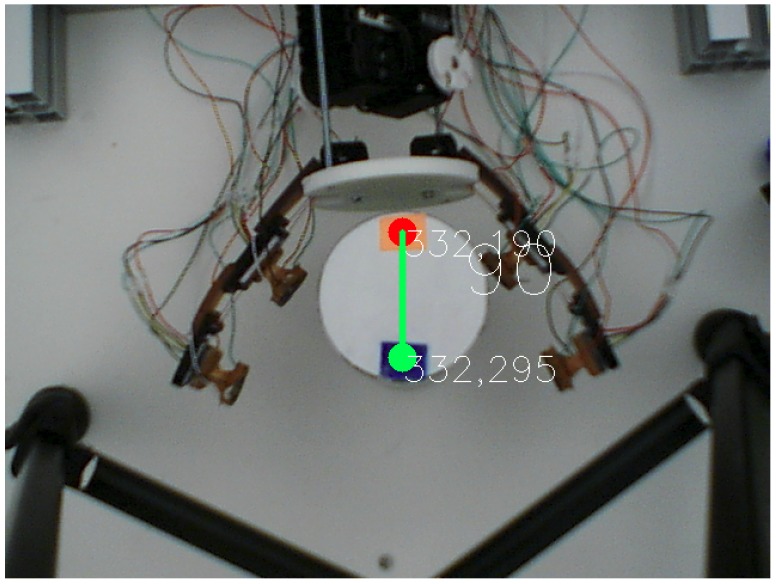
Angle measured from the center of color blobs in the diameter of the object.

The top view of gripper during manipulation was used to estimate object pose variations. The middle of the USB camera image established a frame of reference fixed at the top of this setup. Inside this working space, the difference of pixels determined the angle between two color papers fixed on the object. Figure 13 presents the object angle based on a python script using OpenCV library [35] to capture in-hand angle changes between an object and the camera’s visual frame of reference.

Using the setup presented in this section, stable autonomous fuzzy grasping of objects allowed angle estimations based on initial visual inference with tactile excitation to further pose estimation. The next section presents the results of estimations using external and internal stimuli.

## 4. Results

This section presents the results of two visuotactile experiments on pose estimation using machine learning algorithms. The first experiment used external forces, while the second performs estimation during an open-loop finger self-actuation to rotate an object. In both experiments, images were used to train machine learning algorithms for the dynamic changes in the object’s pose. During all operations, a dual fuzzy controller performs an autonomously maintained stable grasp.

### 4.1. Rotation Using External Forces

**Figure 14:**
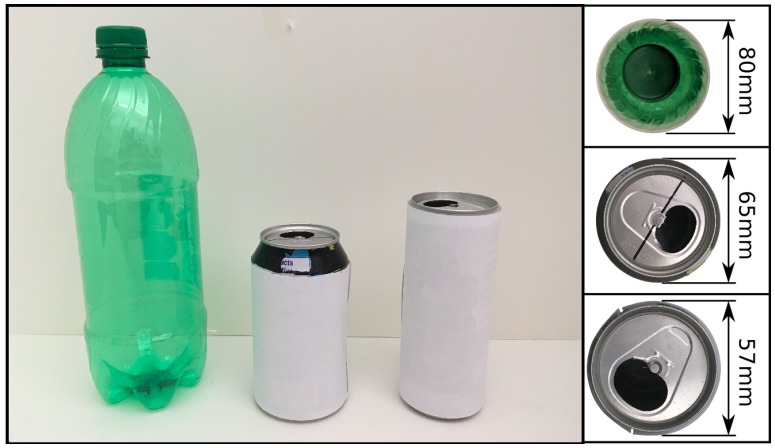
Object used during the first experiment.

During data collection for this first experiment, a human operator executed rotations on a previously grasped object. Figure 14 shows three objects used during this first experiment and its respective diameter information.

Different diameter sizes were used to test the force consistency provided by the autonomous fuzzy controller even with different objects, while still allowing smooth rotation. A piece of paper with a color scheme on object top plate extremities provides a visual cue of the angle to be estimated.

**Figure 15:**
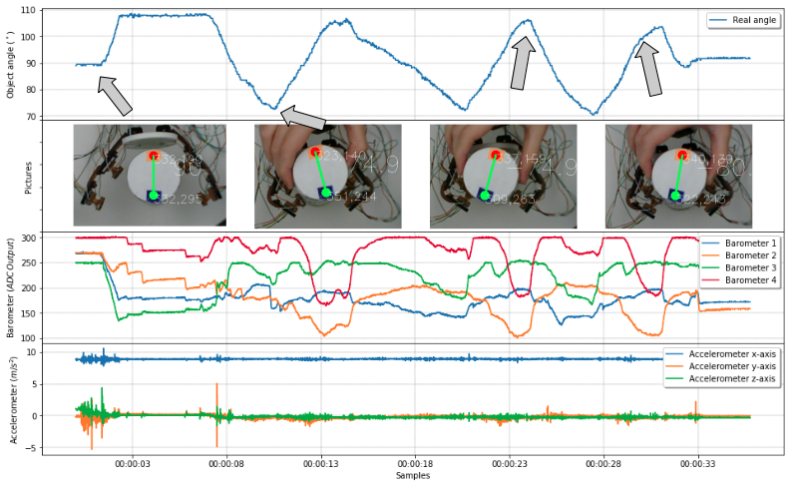
Object being rotated with sensors varying in accordance with the angle.

Figure 15 shows an example of sensor data collection during rotation using external forces. The angle captured by the camera shows on the first row the execution of clockwise and counterclockwise rotations, at least three times each way, which made sure that sensors were excited with all workspace possibilities for this limited setup. A set of pictures on the second row shows the human operator rotating an object, trying to promote tactile sensor exploration of the gripper workspace. Figure 15 also presents pressure information on the third row showing sensor changes while the object pose is changing. The fourth row presents the x-axis of accelerometer data, which has less amplitude but is essential to define the direction of action, information that pressure alone is not capable of providing.

### 4.2. Machine Learning Results

In our visuotactile approach, this stage is where the robot has its camera pointed to the object, learning an egocentric frame of reference. Later, it could free the camera to find an allocentric frame of reference, reallocating its attention, and use only tactile information to feel any object pose changes. From grasping to rotations, we used five executions for each object with 70% of the randomized data for training. Table 3 displays the results, with the ridge regressor outperforming all other algorithms in general for the normalized angle. Denormalizing the angle, the root of the average mean squared error for all sizes for the Ridge Regressor was $1.82^{\circ }$.

**Figure 16:**
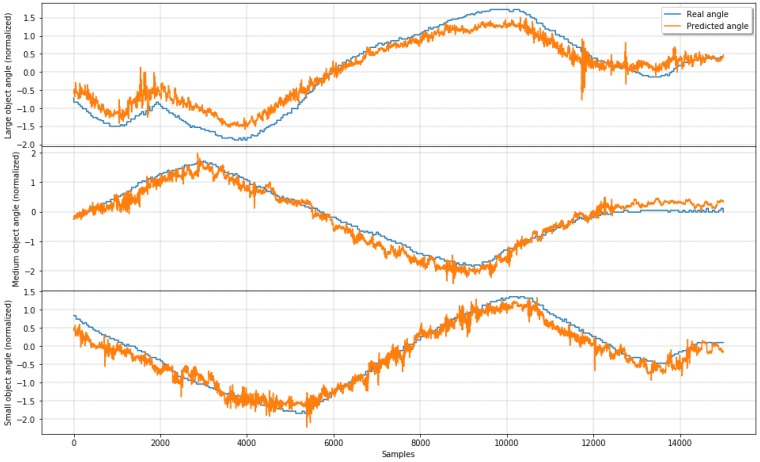
Angle prediction compared to real angle using ridge regression.

Figure 16 shows extracts of real data and angle estimations using ridge regression for each of the three objects used in this experiment. Large, medium, and small diameter objects estimated data with the real angle on each row are shown from top to bottom, respectively. On the second and third lines, angle estimations for medium and small diameter objects show high accuracy for all test data presented to the ridge regressor.

It is possible to observe that the large diameter object results on the first row show slightly less precision, with the predicted line not following the real angle line on some occasions. That observation is compatible with Table 3, where this object shows a mean squared error (MSE) of 0.173, while medium and small objects show 0.159 and 0.077, respectively, for the ridge regressor, while achieving a better result using a neural network.

Log files (rosbags) contained angle information at 30 frames per second, with sensor data collected at its maximum frame rate. Table 3 presents results from five methods, namely: Linear regression, K-nearest neighbors regression (KNN), support vector regression (SVR), ridge regressor, and a neural network. The KNN used five neighbours and the Minkowski metric; SVR trained with the RBF kernel and degree 3; the ridge regressor was trained using α=0.5; moreover, the MLP neural network contained a single hidden layer with one hundred neurons. All method predictions used the same data features: Accelerometer, barometer, and magnetometer. Method accuracy used three metrics: Mean squared error (MSE), coefficient of determination ($R^{2}$), and explained variance regression score (EXP) [36,37,38,39].

### 4.3. Results from Open-Loop In-Hand Manipulation Angle Estimation

**Figure 17:**
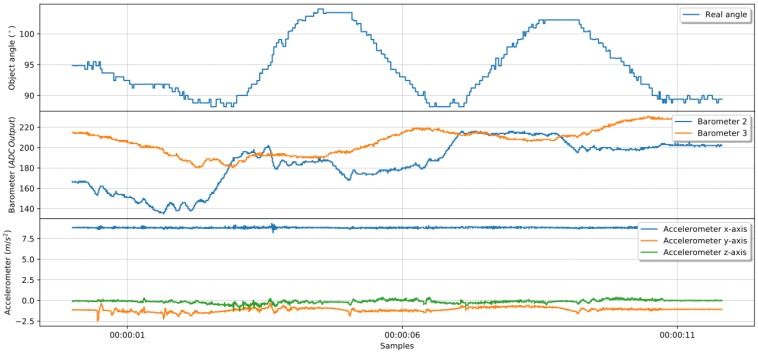
Object being rotated with sensors varying in accordance with the angle.

During the second experiment, one finger performed in-hand manipulations, rotating the object while it was under a stable grasping. Figure 17 presents data collected during an open-loop self-rotation experiment with the object’s angle in the first graph, followed by a data sensor on subsequent lines.

On the first row, it is possible to observe in-hand manipulations promoted by the finger changes the object angle four times. Compatible pressure data are in the second row, with both barometer measurements changing accordingly to object angle as expected. One could also observe changes in accelerometer data presented in the third row.

**Figure 18:**
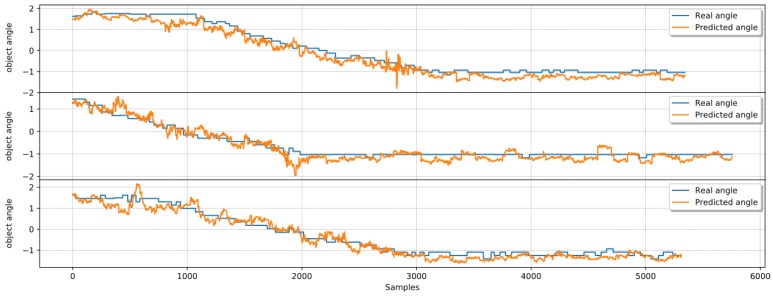
Prediction of angle compared to real angle using Multilayer perceptron (MLP) during self object rotation.

Table 4 presents the results of three experiments where MLP outperforms all other methods during three in-hand self-rotations of a middle-size diameter object. Figure 18 shows extracts of real data and the predicted angle using MLP during self-rotation experiments. All the machine learning techniques mentioned on Section 4.2 after retraining show similar results for the middle-size container used in the three experiments.

It is possible to see that estimations for angle during all three experiments shown in the three rows are following not only the tendency of the real angle measurement but achieving reasonable precision for all test data presented to the neural network. Observation is compatible with data from Table 4, which shows MLP results with MSE of 0.77, 0.051, and 0.067 for the first, second, and third experiments, respectively. In the next section, we draw some conclusions from the results of the experiments presented here.

## 5. Discussion

Underactuated hands are a useful tool for in-hand manipulation tasks due to their capability to seamlessly adapt to the contour surfaces of unknown objects and maintain such objects under grasp even when disturbed by external stimuli. These hands are incredibly versatile while grasping unknown objects, but after grasping, estimating the pose of in-hand objects becomes a challenge due to the flexibility of the fingers in such devices. To reduce the pose uncertainty of in-hand objects, we developed a visuotactile approach inspired by the human somatosensory system.

The human manipulation system operates with two subsystems with the “Where” system dealing with perception to action building egocentric and allocentric frames of reference [5]. The present paper introduced a visuotactile approach for robotic in-hand pose estimation of objects. In this research, it was used as inspiration for after grasping object pose estimation combining visual and tactile sensing information. During the experiments, autonomous, stable grasping using a dual fuzzy controller provides consistent force even while handling different objects. Two sets of experiments achieved accurate angle estimation using tactile data.

The system proposed in this paper is an attempt to emulate the human “Where” subsystem. It explores machine learning methods to find the relationship between the data collected by multimodal tactile sensing modules and the orientation of an object under grasp given by intermittent allocentric reference frames. The main advantage of such a data-driven approach is that it could extend to different gripper configurations with more sensing modules and objects with different shapes.

In the first experiment, external forces were applied to the object during a stable grasping, forcing it to change its orientation, while during the second experiment, autonomous open-loop promoted object rotation. External stimuli promote learning of some representations between tactile sensing inputs an object poses, which sometimes are not achieved by a robots finger workspace. Controlled pose disturbances during in-hand manipulation also promote robot learning of some representations between tactile sensing inputs and object poses a robot may need to be exposed to, which can be achieved through the workspace of its fingers. After an initial visual exploration, angle change estimation uses tactile data from bio-inspired sensor modules. Post-processing with ridge regressor achieved an average mean squared error of 0.077 during experiments using external forces. Compatible results using an MLP neural network achieved a mean squared error of 0.067 during autonomous open-loop rotation in the second experiment. As robots become more complex and present in unstructured environments, they have to deal with unexpected object manipulation scenarios. Being able to use its camera in conjunction with tactile sensing is essential to develop a sustainable robot presence in the modern world.

Future research could focus on integration with the somatosensory system in real-time object recognition and pose estimation. Robots that achieve a reasonable level of dexterity using the allocation of attention will improve the efficiency of use of its resources to perform a broader range of tasks in unstructured environments. Since the solution is model-free, the selected machine learning algorithms will improve its accuracy as more information is available, which will lead the investigation of this approach using a more significant number of fingers. A simple pick and place task where a table is modified from its initial position would be improved in this scenario. An “egocentric” frame of reference related to tactile sensing modules is created after exploring the object with the visuotactile system. This robot could build an “egocentric” frame of reference centered tactile control system, while a now free vision system will find a “allocentric” frame of reference (e.g., the axis of a table) for interobject interaction.

## Figures and Tables

**Table 1:** Finger fuzzy feedback controller rulebook.

	Presssure	No	Low	Normal	High
Microvibrations					
Low		NT/S	T/S	H/S	P/S
High		NT/NS	T/NS	NS	P/NS

**Table 2:** Grasp fuzzy controller rulebook.

	Finger 2	NT	T	H	HS	HNS	PS
Finger 1							
NT		GF1/GF2	H1	GF1	GF1	GF1	H2
T		H1	GF1/GF2	H2			H2
H		GF2	H1			GF1/GF2	H2
HS		GF2			H1/H2		H2
HNS		GF2		GF1/GF2			H2
PS		H1	H1	H1	H1	H1	GB1/GB1

**Table 3:** Angle estimation using external forces with blue indicating best results.

Regressor	Large			Medium			Small		
	MSE	$R^{2}$	EXP	MSE	$R^{2}$	EXP	MSE	$R^{2}$	EXP
Linear Regression	0.173 (0.13)	0.825 (0.12)	0.903 (0.02)	0.159 (0.12)	0.836 (0.11)	0.898 (0.04)	0.077 (0.05)	0.898 (0.05)	0.946 (0.002)
K-Nearest Neighbors	0.289 (0.15)	0.691 (0.16)	0.760 (0.09)	0.515 (0.54)	0.501 (0.41)	0.683 (0.18)	0.102 (0.02)	0.859 (0.03)	0.899 (0.02)
Support Vector Regression	0.199 (0.12)	0.794 (0.11)	0.874 (0.04)	0.228 (0.15)	0.762 (0.11)	0.829 (0.11)	0.116 (0.03)	0.836 (0.06)	0.892 (0.01)
Ridge Regression	0.173 (0.13)	0.825 (0.12)	0.903 (0.02)	0.159 (0.12)	0.836 (0.11)	0.898 (0.04)	0.077 (0.05)	0.898 (0.05)	0.946 (0.002)
Neural Network	0.171 (0.10)	0.821 (0.09)	0.890 (0.05)	0.240 (0.13)	0.743 (0.09)	0.864 (0.07)	0.085 (0.02)	0.880 (0.04)	0.925 (0.007)

**Table 4:** Self-rotation angle estimation with blue indicating best results.

Regressor	Exp 1			Exp 2			Exp 3		
	MSE	$R^{2}$	EXP	MSE	$R^{2}$	EXP	MSE	$R^{2}$	EXP
LR	0.175	0.858	0.958	0.524	−0.155	0.781	0.531	0.492	0.743
KNN	0.389	0.686	0.686	0.203	0.552	0.553	0.193	0.814	0.818
SVR	0.218	0.824	0.830	0.107	0.762	0.812	0.268	0.743	0.770
RIDGE	0.175	0.858	0.958	0.524	−0.154	0.78	0.531	0.493	0.744
MLPR	0.077	0.937	0.976	0.051	0.885	0.898	0.067	0.936	0.946
